# Editorial: Non-coding RNAs: insights and state-of-the-art in gastrointestinal sciences

**DOI:** 10.3389/fphys.2023.1248855

**Published:** 2023-07-05

**Authors:** Ting Fu, Zhenjiang Zech Xu, Cuncong Zhong

**Affiliations:** ^1^ Pharmaceutical Sciences Division, School of Pharmacy, University of Wisconsin Carbone Cancer Center (UWCCC), University of Wisconsin–Madison, Madison, WI, United States; ^2^ State Key Laboratory of Food Science and Technology, Nanchang University, Nanchang, China; ^3^ Department of Electrical Engineering and Computer Science, University of Kansas, Lawrence, KS, United States

**Keywords:** noncoding RNA, gastrointestinal disease, biomarker, cancer biology, early diagnoosis

Gastrointestinal diseases encompass a wide range of physiological disorders such as gastroesophageal reflux disease (GERD), peptic ulcers, inflammatory bowel disease (IBD), irritable bowel syndrome (IBS), liver diseases (such as hepatitis and cirrhosis), and gastrointestinal cancers (GC). Every year, hundreds of millions of individuals suffer from one or more of these diseases globally, severely affecting the wellbeing of human life and challenging our healthcare system. Noncoding RNA (ncRNA) refers to a class of RNA molecules that do not encode proteins; instead, they play critical regulatory, structural, or functional roles within cells. Typical classes of ncRNA include microRNA (miRNA), circular RNA (circRNA), ribozyme, long noncoding RNA (lncRNA), etc. In this Research Topic, we are excited to present four outstanding review/opinion articles discussing how ncRNAs are involved in various types and stages of gastrointestinal diseases, with an emphasis on ncRNA’s clinical applications such as early detection, progression monitoring, treatment response prediction, and serving as therapeutic targets.

Sato et al. presented a comprehensive discussion of the roles of lncRNAs and their subclass circRNAs in pancreatic ductal adenocarcinoma (PDAC). PDAC develops from normal ducts in the pancreas and its altered precancer lesion, pancreatic intraepithelial neoplasia (PanIN). Epithelial-mesenchymal transition (EMT) is a critical pathophysiological step that associates PDAC cells with invasion, migration, and metastasis. During EMT, epithelial cells lose their cell polarity and cell-cell adhesion and gain migratory and invasive properties to become mesenchymal cells. Recently, lncRNAs and circRNAs have been characterized to participate in EMT in PDAC, which can affect the migration and invasion of tumor cells. LncRNAs can act as competing endogenous RNAs (ceRNA) to sequester target miRNAs, bind to spatially close genes, and directly control EMT-related proteins. For example, the HOX transcript antisense RNA (HOTAIR) can directly bind to or indirectly interact with polycomb repressive complex 2 (PRC2), which further mediates chromatin regulation in EMT and PDAC progression. CircRNAs mostly regulate the EMT process in PDAC by acting as a miRNA sponge, directly affecting the protein degradation process. For instance, circ-NEIL3 regulates adenosine deaminases, which act on the RNA 1 (ADAR1) expression by sponging miR-432-5p to induce RNA editing of glioma-associated oncogene 1 (GLI1), ultimately influencing cell cycle progression and promoting EMT in PDAC cells. Given the important functions of lncRNA and circRNA in PDAC, Sato et al. further discussed the potential of using these ncRNAs as biomarkers for the early detection of PDAC.

Gareev et al. investigated the potential of using circulating ncRNAs (including lncRNA, miRNA, and circRNA) in gastric juice as non-invasive biomarkers for GC monitoring and treatment. Mechanistically, ncRNAs can participate in critical biological processes during GC oncogenesis, including cell proliferation, migration, invasion, apoptosis, differentiation, angiogenesis, and metastasis. As a result, dysregulated ncRNAs in GCs can act as oncogenes or tumor-suppressors in cancer progression. Noncoding RNAs may be secreted outside the cell via extracellular vesicles (EVs), exosomes, and microvesicles. The so-called circulating ncRNA tends to be highly stable in biological fluids and is resistant to endogenous ribonucleases and adverse physical conditions, allowing us to probe the physiological conditions of the cells via profiling these circulating ncRNAs. Technically, gastric juice has a higher concentration of EVs, originating from tumor cells and surrounding cells, compared to those in blood. In addition, gastric juice did not contain ribonucleoproteins and lipoprotein complexes, which inevitably contaminates EV in blood. Both factors make gastric juice circulating ncRNAs ideal non-invasive biomarker candidates for use in GC screening.

Usman et al. focused on the mechanism and clinical applications of using ncRNA as a biomarker for determining GC radiotherapy response. They highlighted the roles of ncRNAs in DNA damage repair, cell apoptosis, and activation of epidermal growth factor receptor signaling pathways, which eventually turn into different responses to radiotherapy. They further pointed out that the above findings could make ncRNAs potential therapeutic targets for developing new GC treatments. While continuing research was needed to fully understand the complex interactions between ncRNAs and other cellular processes involved in regulating the response to GC radiotherapy, this article provided a comprehensive overview of current research in this field and emphasized the clinical value of ncRNA. Despite the great potential, Usman et al. also highlighted the need for continuing research in this area to further improve treatment outcomes for GC patients.

In addition to focusing on reviewing ncRNA’s potential as a biomarker, Wusiman et al. further looked into aminoacyl-tRNA synthetases (ARSs) pathophysiology in digestive system diseases, including IBD, liver disease, and colorectal cancer. ARSs are essential enzymes for protein biosynthesis in all cells, and their malfunction has been linked to various pathological conditions. Wusiman et al. presented the mechanisms by which ARSs contribute to tumorigenesis and angiogenesis in the digestive system. They also highlighted potential ARSs-targeted therapies such as via small molecule inhibitors and RNA interference. Overall, this review has provided a comprehensive summary of the current understanding of ARSs’ role in digestive system diseases and called for ongoing research into these enzymes’ functions to unleash their clinical potential.

Continuing investigation of ncRNA mechanisms in gastrointestinal diseases and the development of novel technologies and therapeutics have improved gastrointestinal disease patients’ access to timely, effective, and inexpensive treatments. Furthermore, we have also witnessed how in-depth mechanistic understanding can lead to more accurate gastrointestinal disease early detection and prevention strategies. While the Research Topic of this Research Topic may be far from comprehensive to summarize the important field of ncRNA research in gastrointestinal disease, we anticipate the articles included here shall promote, enlighten, and spark related research in both basic science and clinical settings. We sincerely thank all of our contributors and hope our readers enjoy this Research Topic!

